# A systematic review of psychosocial interventions to improve quality of life of people with cancer and their family caregivers

**DOI:** 10.1002/nop2.543

**Published:** 2020-06-28

**Authors:** Israel Gabriel, Debra Creedy, Elisabeth Coyne

**Affiliations:** ^1^ School of Nursing and Midwifery Griffith University Logan Campus Queensland Australia

**Keywords:** family caregivers, intervention, people with cancer, psychological, psychosocial, quality of life, spirituality, systematic review

## Abstract

**Aims:**

To review the characteristics and effectiveness of psychosocial interventions on quality of life of adult people with cancer and their family caregivers.

**Design:**

A systematic review using PRISMA guidelines.

**Methods:**

Seven databases were searched from 2009–2019 using key terms. Included studies were assessed using the Quality Assessment Tool for Quantitative Studies.

**Results:**

1909 studies were retrieved with 12 studies included, involving 3,390 patients/caregivers. Interventions aimed to improve communication, behaviour change and setting short‐term goals. Duration of interventions varied from 4–17 weeks. Highest benefit was gained from telephone interventions. Interventions based on interpersonal counselling appeared more effective than other approaches. Studies predominantly focused on psychological, physical and social domains of quality of life. Spiritual well‐being received relatively little attention. A paradigm shift is needed to develop psychosocial interventions that incorporate spiritual well‐being. More research is needed in developing countries.

## 1 INTRODUCTION

People with cancer and their families often experience a decline in health‐related quality of life (QoL) following diagnosis (Nayak et al., 2017). According to the World Health Organization (1998:551), QoL is an “individual's perception of their position in life in the context of the culture and value systems where they live and in relation to their goals, expectations, standards and concerns.” Patients and their family caregivers are profoundly affected by the cancer experience and often respond to cancer as a combined system (Coyne, Dieperink, Østergaard, & Creedy, 2017). Griffin et al. (2014:1,275) defined family caregivers as “those, related and non‐related, who provide direct care and support to people living with cancer.” Both patients and their family caregivers may experience frequent adverse physical and psychological symptoms (Bonacasa, Rosa, Camps, & Martínez‐Rubio, 2019; Johansen, Cvancarova, & Ruland, 2018), and social and spiritual difficulties (Bai, Brubaker, Meghani, Bruner, & Yeager, 2018; Grégoire et al., 2017).

In recent years, QoL has been accepted as an important outcome criterion when evaluating the effectiveness of oncology treatments (Sibeoni et al., 2018). The multidimensional construct of QoL comprises four components: psychological, physical, social and spiritual (Janz et al., 2014). The psychological domain includes emotional well‐being related to memory and positive and negative indicators of mood (e.g. stress depression symptoms). Psychological impairment can restrict individuals' perceptions of their health and coping. The physical domain relates to health and physical performance (e.g. pain, fatigue, incontinence). The social domain generally relates to the effects of the disease or caregiving on social and personal roles and perceptions of social support (Yanez, Thompson, & Stanton, 2011). Spiritual well‐being includes coping with life stressors and attainment of self‐transcendence (Counted, Possamai, & Meade, 2018). Spirituality differs from religiosity which usually refers to institutionalized religious beliefs (Counted et al., 2018).

In the last 10 years, researchers have developed various psychosocial interventions to improve the QoL of both adult people with cancer and their family caregivers (Ferrell & Wittenberg, 2017; Hu, Liu, & Li, 2019). However, most research has predominantly focused on psychological, physical and social domains (Gabriel & Mayers, 2019; Titler et al., 2017). Furthermore, several published systematic reviews and meta‐analyses (Badr & Krebs, 2013; Fu, Zhao, Tong, & Chi, 2017; Son, Son, Kim, & Lee, 2018) that evaluated the effects of psychosocial interventions on QoL have limitations. For example, the review by Fu et al. (2017) reported on general QoL, instead of dimensional QoL scores, and only focused on cancer family caregivers.

Spiritual well‐being has received relatively little attention despite its influence on QoL (Skalla & Ferrell, 2015). Distress over spiritual concerns has been found to be prevalent among people with cancer (Drummond & Carey, 2019). Badger et al. (2011) who examined the effectiveness of a brief telephone psychosocial intervention with seventy‐one prostate cancer survivors and their family partners found spiritual well‐being was an important predictor of QoL. Other studies report that spiritual well‐being contributes to better health outcomes (Lichter, 2013; MacKinlay & Burns, 2017). Limiting psychosocial interventions to psychological, physical or social domains may provide inadequate information on the applicability and effect of such interventions.

Most intervention studies have been conducted in developed countries such as the United States of America (USA), Australia, Canada or France. Although the overall incidence of cancer is higher in developed countries, total cancer‐related mortality is significantly higher in developing (low‐ and middle‐income) countries, where 75% of cancer deaths occur and the number of cancer cases is rising most rapidly (Prager et al., 2018). In African society for example, cancer is still considered incurable (Wallace, Bos, & Noble, 2018). Although people with cancer and their families are reported to seek positive meaning for their circumstances through spiritual endeavours (Kiyancicek & Caydam, 2017), the extent to which this is the case is unknown. To address this apparent gap and inform future work in low‐ and middle‐income countries, there is a need to critical appraise the effectiveness of possible interventions on the four domains of QoL for both people with cancer and their family caregivers.

## 2 AIM

The aim was to review the characteristics and effectiveness of psychosocial interventions on QoL of adult people with cancer and their family caregivers. The research questions were as follows: (a) What are the characteristics of psychosocial interventions implemented with adult people with cancer and their family caregivers in developed and developing countries? (b) How effective are these psychosocial interventions on QoL of adult people with cancer and family caregivers in developed and developing countries?

## 3 METHODS

The review process was guided by the Preferred Reporting Items for Systematic Review and Meta‐Analysis (PRISMA) recommended protocols (File S1). The PRISMA checklist is an evidence‐based tool for evaluating the title, abstract, methods, results, discussion and findings. It can be used for evaluating randomized controlled trials and reporting systematic reviews for non‐heterogeneous research (Liberati et al., 2009). The review protocol is registered with PROSPERO CRD42020144563.

### 3.1 Search strategy

Original articles were identified using databases, including PubMed, MEDLINE, CINAHL, PsycINFO, Web of Science, World Health Organization International Clinical Trials Registry and the International Standard Randomised Controlled Trial Number Registry. The search was limited to articles published in English between 2009–2019. The search terms were combinations of the following keywords: psychosocial therapy OR psychosocial intervention OR education OR counselling OR behavioural therapy AND cancer patient OR neoplasm OR tumour AND caregiver OR family caregiver OR informal caregiver OR primary caregiver AND quality of life OR well‐being OR spirituality OR spiritual well‐being. The search strategy was modified as appropriate for different databases. A hand search of all included studies' reference lists was conducted to identify any relevant studies.

### 3.2 Study selection

The selection process for eligible studies was based on specified inclusion/exclusion criteria (Table 1). All references were downloaded into Endnote version 9, and titles were screened in Endnote by two reviewers. A second check of all retrieved data was undertaken by the main author and second reviewer. All studies were screened first according to title and then abstract and reviewed by two independent reviewers for inclusion. Disagreements were resolved through consensus.

**TABLE 1:** Inclusion and exclusion criteria for searching

	Inclusion criteria	Exclusion criteria
Study design	Intervention studies Randomized and non‐randomized Designs	Unpublished papers Qualitative studies Studies not in English
Population	Studies that involved: adult people with cancer and their family caregivers	Studies that focused on people with cancer or family caregivers only; included nurses and other health professionals
Intervention	Psychosocial interventions that involved: Psychological support (cognitive‐behavioural therapy, psychotherapy counselling, supportive therapy) Social support (social‐skill training) Interventions delivered by trained personnel such as nurses, social workers or other health workers	Interventions did not involve behavioural therapy, psychological support or social support
Outcomes	QoL: psychological/emotional; physical, social; or spiritual domains	Outcomes that did not include QoL. Studies that did not use formal psychometric scales to assess QoL

### 3.3 Quality assessment of included studies

Included studies were assessed for quality by three researchers using the quality assessment tool for quantitative studies (QATFQS) including randomized and non‐randomized designs (Thomas, Ciliska, Dobbins, & Micucci, 2004). The QAFTQS contains eight components of study quality related to sample selection, study design, identification and treatment of confounders, blinding of outcome assessors and of participants, reliability and validity of data collection methods, withdrawals and dropouts, and intervention integrity and analyses. Each component is rated as strong, moderate or weak. Studies with at least four strong ratings are considered strong, less than four strong ratings and one weak rating are considered moderate and two or more weak ratings are considered weak (Thomas et al., 2004).

### 3.4 Data extraction

Relevant information was extracted and recorded in a spreadsheet using Microsoft Excel software. A second check of all retrieved data was undertaken by the main author and second reviewer. Twelve full‐text articles were extracted and tabulated. Extracted data included general information (first author, place of study, publication year, aims and theoretical approach), methodological information (study design, sample context, response rate, follow‐up, retention, therapy type, intervention delivery/dosage and intervention/control group content and measurement tools) and results of the study (main outcomes).

### 3.5 Data analysis

According to the Cochrane Collaboration (Deeks, Higgins, & Altman, 2008), the analysis of findings can be presented as a narrative such as a summary with a discussion of study characteristics and findings. The high degree of methodological diversity, statistical and clinical heterogeneity of included studies did not afford an opportunity to pool results and conduct a meta‐analysis. Thus, a narrative account is presented as an overview of psychosocial interventions for adult people with cancer and family caregivers. Descriptive statistics describe participant characteristics and results.

### 3.6 Search process

**FIGURE 1:**
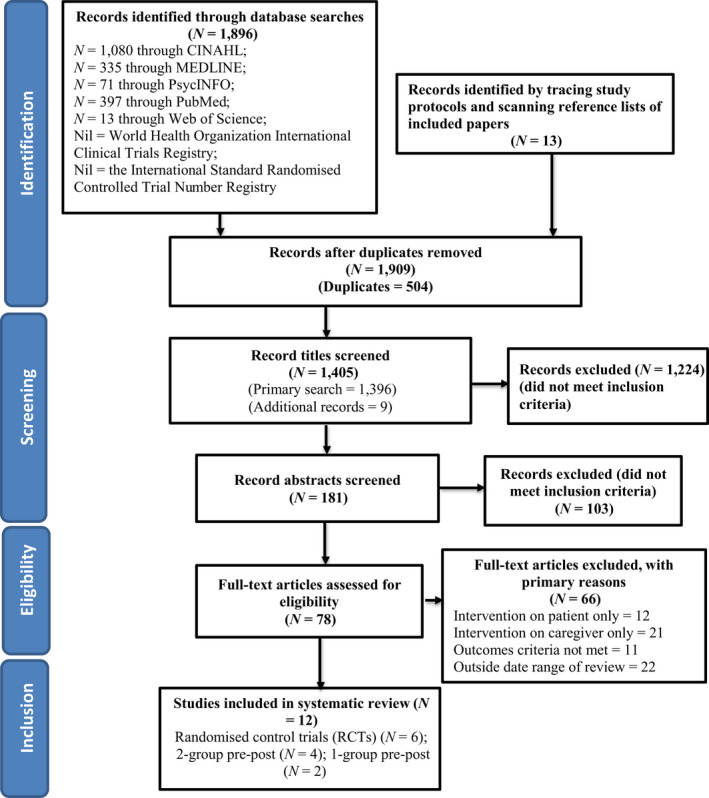
PRISMA flowchart of article inclusion and exclusion

Seven databases were searched for the period from 2009–August 2019. A total of 1909 studies were identified. Duplicate studies were removed (*N* = 504) and those studies that did not meet inclusion criteria were excluded (*N* = 1,327). Full‐text studies (*N* = 78) were checked according to the inclusion/exclusion criteria, resulting in 66 studies being removed leaving 12 studies for full review as described in the PRISMA flowchart (see Figure 1). Reasons for exclusion included qualitative studies only, focus on patients or caregivers only, studies of nurses and other health professionals, did not measure QoL as an outcome or not published in English (Table 1).

### 3.7 Summary of included studies

Results of the quality appraisal process are presented according to PRISMA reporting guidelines. This includes study selection, study characteristics, risk of bias in individual studies and results of individual studies.

Ten studies were conducted in the United States (Badger et al., 2011; Badger, Segrin, Hepworth, et al., 2013; Badger, Segrin, Pasvogel, & Lopez, 2013; Clark et al., 2013; Kayser, Feldman, Borstelmann, & Daniels, 2010; Meyers et al., 2011; Mosher et al., 2018; Northouse et al., 2013, 2014; Titler et al., 2017), one in France (Belgacem et al., 2013) and Australia (Shaw et al., 2016). There were no studies from developing countries.

### 3.8 Risk of bias

As assessed by the QATFQS checklist (see Table 2), risk of bias showed mixed quality: one study (Northouse et al., 2013) was ranked as strong, two studies were evaluated as moderate quality (Clark et al., 2013; Shaw et al., 2016) and nine studies were weak (Badger et al., 2011; Badger, Segrin, Hepworth, et al., 2013; Badger, Segrin, Pasvogel, et al., 2013; Belgacem et al., 2013; Kayser et al., 2010; Meyers et al., 2011; Mosher et al., 2018; Northouse et al., 2014; Titler et al., 2017). Poor quality related to two issues, selection bias and blinding. Common weaknesses of the studies were confounders such as high withdrawal rate or drop out and lack of a control group (Northouse et al., 2014; Titler et al., 2017). Three studies used randomization but did not describe the process (Badger, Segrin, Hepworth, et al., 2013; Badger, Segrin, Pasvogel, et al., 2013; Mosher et al., 2018). Although most studies were assessed as weak, their consistent findings contribute to our understanding of research in this area and are included in the results.

**TABLE 2:** Quality assessment of the included studies using QATFQS

	Badger, Segrin, Hepworth et al. (2013)	Badger, Segrin, Pasvogel, and Lopez (2013)	Badger et al. (2011)	Belgacem et al. (2013)	Clark et al. (2013)	Kayser et al. (2010)	Meyers et al. (2011)	Mosher et al. (2018)	Northouse et al. (2014)	Northouse et al. (2013)	Shaw et al. (2016)	Titler et al. (2017)
Selection bias	3	3	3	3	1	3	3	3	3	2	2	2
Study design	3	3	3	1	1	1	1	3	3	1	1	3
Confounders	2	3	2	2	2	1	1	2	3	1	1	3
Blinding	3	3	3	3	3	3	3	2	2	2	3	3
Data collection method	1	1	1	1	1	1	1	1	1	1	1	1
Withdrawals and dropouts	2	2	1	1	1	2	3	2	1	2	1	1
Intervention integrity	1	2	1	2	1	1	1	1	1	1	1	1
Analyses	1	1	1	1	1	1	1	1	1	1	1	1
Total	3	3	3	3	2	3	3	3	3	1	2	3

Note

Key: strong = 1; moderate = 2; weak = 3.

### 3.9 Study characteristics

#### 3.9.1 Participants

The 12 included studies addressed adult cancer patients and their family caregivers as a dyad. The overall total number of participants enrolled at baseline was 3,390 patients/caregivers, but sample sizes varied considerably between studies from 80 (Titler et al., 2017)–968 (Northouse et al., 2013). Only two studies had sample sizes <100 (Northouse et al., 2014; Titler et al., 2017). The mean age of patients/survivors ranged from 47–67, and between 43–61 years for family caregivers. The mean response rate of patient/caregiver dyads was 55.3% (range = 14% (Kayser et al., 2010)–93% (Clark et al., 2013)). At final follow‐up, the mean attrition rate was 22.9% (range = 0% (Belgacem et al., 2013)–71% (Meyers et al., 2011)).

Cancer diagnosis of patient/survivors included breast cancer (Badger, Segrin, Hepworth, et al., 2013; Badger, Segrin, Pasvogel, et al., 2013; Kayser et al., 2010), prostate cancer (Badger et al., 2011) and a mix of cancer diagnoses (Belgacem et al., 2013; Clark et al., 2013; Meyers et al., 2011; Mosher et al., 2018; Northouse et al., 2013, 2014; Shaw et al., 2016; Titler et al., 2017). Family caregivers included spouses or significant other, child, sibling, parent, other relative or close friend. While one study (Kayser et al., 2010) focused exclusively on spousal caregivers, other studies took a broader approach and included the patient's nominated family caregiver.

Interventions included interpersonal counselling and health education, skills training and coping skills, family connection interventions, the FOCUS (Family involvement, Optimistic attitude, Coping effectiveness, Uncertainty reduction and Symptom management) programme, or COPE (Creativity, Optimism, Planning and Expert information) programme (Meyers et al., 2011) targeting people with cancer and their family caregivers (as described in Table 3).

**TABLE 3:** Summary of systematic review of studies on psychosocial interventions

Author (year), place	Sample size, age (mean), cancer type	Study design response rate, and attrition	Follow‐up	Measures	Intervention (include duration)
Badger, Segrin, Pasvogel, and Lopez (2013), USA	Intervention 1 (*N* = 36), intervention 2 (*N* = 40), intervention 3 (*N* = 28) Mean age: 52 Cancer type: breast	2‐group pre‐post Response rate: 41% Attrition rate: 23%	8 weeks	CES‐D, GSDS, SWSQOLC, SWSQOLC	8‐week IC/VC or THE; 30 min/week Delivered by social worker
Badger, Segrin, Hepworth, et al. (2013), USA	Intervention 1 (*N* = 90), intervention 2 (*N* = 90). Age: 43 Cancer type: breast	2‐group pre‐post Response rate: 50% Attrition rate: 22%	8 weeks	CES‐D, GSDS, SWSQOLC, SWSQOLC	8‐week TIP‐C or THE; 29 min/week Delivered by social worker
Badger et al. (2011), USA	Intervention 1 (*N* = 72), intervention 2 (*N* = 70). Age: 62 Cancer type: prostate	2‐group pre‐post Response rate: 43% Attrition rate: 10%	8 weeks	CES‐D, MFI, SWSQOLC, SWSQOLC	8‐week TIP‐C or HEAC; 29–31 min/week Delivered by nurse or social worker
Belgacem et al. (2013), France	Intervention (*N* = 66), control (*N* = 68). Age: 57 Cancer type: haematological and others	RCT Response rate: 43% Attrition rate: Nil	No	SF36 Health Survey	Caregiver educational programme Control group: standard care Delivered by nurses
Clark et al. (2013), USA	Intervention (*N* = 130), control (*N* = 128). Age: 59 Cancer type: mixed	RCT Response rate: 93% Attrition rate: 20%	27 weeks from baseline	FACT‐G, CQOLC, FACIT‐Sp	4‐week SMI 90 min Control group: standard medical care. Delivered by clinical psychologist, nurse, chaplain and social worker
Kayser et al. (2010), USA	Intervention (*N* = 54), control (*N* = 72). Age: 46 Cancer type: breast	RCT Response rate: 14% Attrition rate: 25%	12 months from baseline	FACT‐B, QL‐SP	17 weeks PICP 60 min/biweekly Control group: SSWS Delivered by social worker
Meyers et al. (2011), USA	Intervention (*N* = 696), control (*N* = 256). Age: 61 Cancer type: mixed	RCT Response rate: 57% Attrition rate: 71%	30 days, 60 days, 90 days, 120 days, 180 days	COH	4‐week COPE Control group: usual care Delivered by health educator
Mosher et al. (2018), USA	Intervention 1 (*N* = 50), intervention 2 (*N* = 50). Age: 57 Cancer type: mixed	2‐group pre–post Response rate: 57% Attrition rate: 22%	6–11 weeks from baseline	FACIT‐Sp, PROMIS,	5‐week TCSI or PPHCSI 60 min/week Delivered by PhD clinical psychology students
Northouse et al. (2014), USA	Intervention (*N* = 88) Age: 54 Cancer type: mixed	1‐group pre–post Response rate: 51% Attrition rate: 14%	No	FACT‐G	6‐week web‐based FOCUS 60 min/biweekly Delivered by nurses
Northouse et al. (2013), USA	Intervention 1 (*N* = 318), intervention 2 (*N* = 324), control (*N* = 326). Age: 57 Cancer type: mixed	RCT Response rate: 69% Attrition rate: 38%	6 months from baseline	FACT‐G	10‐week FOCUS 30–90 min/week Control group: usual care Delivered by nurses
Shaw et al. (2016), Australia	Intervention (*N* = 130), control (*N* = 128) Age: 56 Cancer type: mixed	RCT Response rate: 75% Attrition rate: 20%	6 months from baseline	Short Form SF‐12 FACT‐G	10‐week FCI Control group: usual care Delivered by clinical psychologists
Titler et al. (2017), USA	Intervention (*N* = 80) Age: 60 Cancer type: mixed	1‐group pre‐post Response rate: 71% Attrition rate: 10%	No	FACT‐G	5‐week FOCUS 120 min/week Delivered by social worker and family therapist

Abbreviations: CES‐D, Centre for Epidemiological Studies‐Depression Scale; COH, City of Hope; COPE, Creativity, Optimism, Planning, and Expert information; CQOLC, Caregiver Quality of Life Index‐Cancer Scale; FACIT‐Sp, Functional Assessment of Chronic Illness Therapy—Spiritual Well‐Being scale; FACT‐B, Functional Assessment of Cancer Therapy—Breast; FACT‐G, Functional Assessment of Cancer Therapy; FCI, The Family Connect Intervention; FOCUS, Family involvement, Optimistic outlook, Coping effectiveness, Uncertainty reduction, and Symptom management; GSDS, General symptom distress scale; HEAC, health education attention condition; HEAC, Health Education Attention Condition; IC, telephone counselling; MFI, Multidimensional Fatigue Inventory; PICP, Partners in Coping Program; PPHCSI, Peer Helping plus Coping Skills Intervention; PROMIS, Patient Reported Outcomes Measurement Information System; QL‐SP, Quality of Life Questionnaire for Spouses; SMI, Structured Multidisciplinary Intervention; SSWS, standard social work services; SWSQOLC, Social Well‐being Subscale of the Quality of Life Cancer instrument; SWSQOLC, Spiritual Well‐being Subscale of the Quality of Life Cancer instrument; TCSI, Telephone‐based Coping Skills Intervention; THE, telephone health education; TIP‐C, telephone interpersonal counselling; VC, video counselling.

Duration of interventions ranged from 4–17 weeks, and session length varied from 29–120 min. Most interventions were delivered in eight sessions; however, the number of sessions ranged from three (Meyers et al., 2011)–nine (Kayser et al., 2010). Delivery of the interventions involved telephone, face‐to‐face and web‐based.

#### 3.9.2 Frameworks or theory

Nine studies were based on specific theoretical frameworks, such as the stress‐appraisal model by Lazarus and Folkman (Northouse et al., 2013, 2014; Titler et al., 2017). Three were guided by cognitive‐behavioural theory (CBT) (Kayser et al., 2010; Meyers et al., 2011; Mosher et al., 2018) and three by interpersonal theory (Badger et al., 2011; Badger, Segrin, Hepworth, et al., 2013; Badger, Segrin, Pasvogel, et al., 2013). Three studies (Belgacem et al., 2013; Clark et al., 2013; Shaw et al., 2016) had no explicit theoretical framework.

Lazarus and Folkman's transactional model of stress and coping evaluates the processes of coping with numerous stressors, such as symptoms, treatment, work stress, family stress and the challenge of caregiving (Lazarus & Folkman, 1987). This has been widely advocated as a useful framework to guide cancer research (Ravindran, Shankar, & Murthy, 2019). Three studies were underpinned by CBT principles. According to Daniels (2015:54), CBT is a “psychotherapeutic approach that emphasizes the significance of how thinking affects the feelings.” CBT may include cognitive restructuring, relaxation and skills training among other modalities. CBT focuses on the present and aims to empower individuals to change their responses to circumstances. Interpersonal theory proposes an evidence‐based, life event and affect‐focused treatment approach based on the premise that distress does not occur in a social vacuum, but is influenced by and affects the patient's psychosocial environment. The goal of interpersonal therapy is to help individuals solve a crisis in role functioning or social environment, which leads to the improvement in QoL (Blanco et al., 2019). This technique has been established as feasible for treating major depressive disorder in patients with breast cancer (Blanco et al., 2014).

Different psychological/psychosocial therapies were used including interpersonal counselling and health education (Badger, Segrin, Hepworth, et al., 2013; Badger, Segrin, Pasvogel, et al., 2013), skills training and coping skills (Kayser et al., 2010; Mosher et al., 2018), FOCUS programme (Northouse et al., 2013, 2014; Titler et al., 2017), COPE programme (Meyers et al., 2011) and family connection interventions, including the assessment of caregivers' needs, family relationship maintenance and self‐care of caregivers (Shaw et al., 2016).

The FOCUS programme is a multicomponent intervention that addresses family involvement, family communication and working on problems as a group. Maintaining an optimistic attitude aims to help the family to keep hope and focus on achieving short‐term goals. Coping effectiveness and uncertainty reduction provide strategies on how to get information and live without doubt; symptoms management emphasizes self‐care strategies to control symptoms and experiences (Tabrizi & Alizadeh, 2018).

The COPE programme (Meyers et al., 2011) is a supportive educational programme, designed to teach cancer patients and family caregivers problem‐solving skills to help manage symptoms and other concerns. It focuses on using creativity (viewing problems as challenges that can be overcome), optimism (focusing on the positive, yet being realistic), planning (developing a sound plan to address problems) and expert information (finding and learning from trustworthy sources) to overcome problems (Tofthagen & Chesak, 2019). Other psychosocial interventions provided a psycho‐educational programme (Belgacem et al., 2013) and a structured multidisciplinary programme (Clark et al., 2013) to assist patients and families to improve their skills in meal support, nursing care, welfare care or symptom management.

#### 3.9.3 Comparison group

Six studies used randomized controlled trial designs to compare outcomes of the intervention group to those receiving standard or usual care (Belgacem et al., 2013; Clark et al., 2013; Meyers et al., 2011; Shaw et al., 2016), or a standard social work service (Kayser et al., 2010). Northouse et al. (2013) used three groups with two groups exposed to brief or extensive versions of the FOCUS programme and one receiving usual care. Four studies compared two groups exposed to different interventions with no control group. These studies compared telephone/video interpersonal counselling or telephone health education/health education attention conditions (Badger et al., 2011; Badger, Segrin, Hepworth, et al., 2013; Badger, Segrin, Pasvogel, et al., 2013), or a telephone‐based coping skills intervention compared with a “peer‐assist” plus coping skills intervention (Mosher et al., 2018).

#### 3.9.4 Outcome measures and timing

Outcomes were measured at two time points in three studies (Belgacem et al., 2013; Northouse et al., 2014; Titler et al., 2017), or three time points in nine studies. Three studies had no follow‐up (Belgacem et al., 2013; Northouse et al., 2014; Titler et al., 2017), and nine studies had one follow‐up. Follow‐up timing ranged from immediate postintervention to 1 year later.

Overall QoL was evaluated with different measurement tools including the Functional Assessment of Cancer Therapy—General (FACT‐G version 4) (Clark et al., 2013; Northouse et al., 2014; Shaw et al., 2016; Titler et al., 2017), Functional Assessment of Cancer Therapy—Breast (FACT‐B) (Kayser et al., 2010), City of Hope Quality of Life (Meyers et al., 2011), Quality of Life Questionnaire for Spouses (QL‐SP) (Kayser et al., 2010), Short Form 12 (SF12) (Shaw et al., 2016) and the Caregiver Quality of Life Index‐Cancer Scale (CQOLC) (Clark et al., 2013). Domains of QoL were also assessed, with a total of 16 different assessment tools being used to assess specific domains (see Table 3 for full details).

### 3.10 Overall QoL results

Six of the 12 included studies reported overall scores of QoL. All included studies reported on psychological/emotional and physical domains, 11 studies reported on the social domain and six on the spiritual domain. Only six studies assessed all domains (psychological, physical, social and spiritual) of QoL (Table 4 for details of domains of QoL). Of the six studies that reported on overall QoL, three reported a significant positive change from baseline to postintervention (Clark et al. (2013) *p* < .02; Northouse et al. (2014) *p* < .05; Titler et al. (2017) *p* < .014). In the remaining three studies, no significant change in overall QoL was observed.

**TABLE 4:** Quality‐of‐life domain outcomes of included studies

Author (year)	Quality of life				
	Overall	Psychological	Physical	Social	Spiritual
Badger, Segrin, Hepworth et al. (2013)	Not measured	Increased significantly (*p* < .001) at 3 time points for dyads in TC/VC or THE	Dyads in both interventions reported significant effects for time (*p* < .01). Greater effect from THE for partners	Significant effect for intervention dyads (*p* < .01).	Survivors reported increased spirituality from T1 to T3 (*p* < .01). Partner reports not analysed due to low reliability
Badger, Segrin, Pasvogel, et al. (2013)	Not measured	Improvement in TIP‐C or THE over time (*p* < .001) for dyads	Dyads in both interventions improved at the three time points (*p* < .001)	Dyads in both interventions had a significant improvement at the three‐times measurement (*p* < .001)	Survivors had no significant effect. Partners had a significant improvement (*p* < .001)
Badger et al. (2011)	Not measured	Dyads in the HEAC condition had an improvement (*p* < .001) compared with dyads in TIP‐C	Dyads in the HEAC condition had an improvement (*p* < .01) compared with dyads in TIP‐C	Dyads in the HEAC condition had an improvement (*p* < .001) compared with dyads in TIP‐C	Dyads in the HEAC condition had an improvement (*p* < .001) compared with dyads in TIP‐C
Belgacem et al. (2013)	Not measured	Only caregiver scores were improved compared with control group (*p* < .008)	Patient and caregiver scores were higher compared with control group (*p* < .044)	Only caregiver score was higher compared with the control group (*p* < .001)	Not measured
Clark et al. (2013)	Patients had an improvement compared with the control group at T2 (4 weeks) (*p* < .02). However, not sustained at follow‐up (27 weeks)	No effect for patients and caregivers	Patients improved compared with the control group (*p* < .01) There was no effect noted on the caregivers	There was no effect of the intervention noted on patients and caregivers	There was no significant effect noted on patients and caregivers
Kayser et al. (2010)	Dyads had higher means than the control group at time 2 and time 3, but no statistically significant	Dyads had higher means than the control group at time 2 and time 3, but no statistically significant	Dyads had higher means than the control group at time 2 and time 3, but no statistically significant differences between the two arms	Dyads had higher means than the control group at time 2 and time 3, but no statistically significant	Not measured
Meyers et al. (2011)	Patients and caregivers had a significant decline across time points	Patients and caregivers had a significant decline across time points	Patients and caregivers had no significant changes	Patients and caregivers had a significant decline across time points	Patients had no significant changes. Caregivers showed significant declines across time points
Mosher et al. (2018)	Not measured	The two interventions had no significant effect on the dyads over time	The two interventions had no significant effect on the dyads	No significant effect on the dyads	No significant effects on the dyads
Northouse et al. (2014)	Dyads had significant improvements (*p* < .05)	Not significant	Dyads had significant improvements (*p* < .05)	Not significant	Not measured
Northouse et al. (2013)	Not measured	Patients had an increase mean, but not significant when compared to the control group Caregivers had a significant group and time effect	Dyads not significant.	Dyads had a significant group and time effect (*p* < .002)	Not measured
Shaw et al. (2016)	No significant difference between groups at 3 or 6 months	No significant difference between groups at 3 or 6 months	No significant difference between groups at 3 or 6 months	Not measured	Not measured
Titler et al. (2017)	The intervention had a positive effect on dyads (*p* < .014)	The intervention had a positive effects on dyads (*p* < 012)	No significant effects	No significant effects	Not measured

Abbreviations: HEAC, health education attention condition; TC, telephone counselling; THE, telephone health education; TIP‐C, telephone interpersonal counselling; VC, video counselling.

### 3.11 Psychological/emotional domain

All studies assessed psychological/emotional well‐being, with four studies reporting a statistically significant improvement in this domain (Badger, Segrin, Pasvogel, et al. (2013) dyads *p* < .001; Badger, Segrin, Hepworth, et al. (2013) dyads *p* < .001; Badger et al. (2011) survivors *p* < .001, partners *p* < .05; Titler et al. (2017) dyads *p* < .34). Two studies (Belgacem et al. (2013) *p* < .008; Northouse et al. (2013) *p* < .01) reported a statistically significant improvement among family caregivers. The remaining six studies reported no significant change in the psychological/emotional domain.

### 3.12 Physical domain

Twelve studies assessed the physical well‐being of both people with cancer and their family caregivers. Five studies reported statistically significantt improvement in the physical domain (Badger, Segrin, Pasvogel, et al. (2013) dyads *p* < .01; Badger, Segrin, Hepworth, et al. (2013) dyads *p* < .001; Badger et al. (2011) dyads *p* < .01; Northouse et al. (2014) dyads *p* < .05; Belgacem et al. (2013) patient *p* = .03, caregiver *p* < .01). One study reported a statistically significant improvement in patient physical well‐being only (Clark et al., 2013; *p* < .01).

### 3.13 Social domain

Social well‐being of people with cancer and their family caregivers was conceptualized as the ability to carry out domestic and family roles and increased interactions with family members, friends and peers (National Academies of Sciences et al., 2016). Four out of eleven studies revealed significantly improved social functioning (Badger, Segrin, Pasvogel, et al. (2013) survivors *p* < .001, partners *p* < .01; Badger, Segrin, Hepworth, et al. (2013) dyads *p* < .001; Badger et al. (2011) dyads *p* < .01; Northouse et al. (2013) dyads *p* < .01). One study reported a significant improvement among family caregivers (Belgacem et al. (2013) *p* < .001), while the remaining six studies reported no significant change in social well‐being.

### 3.14 Spiritual domain

Six studies evaluated changes in spiritual well‐being postintervention. A significant improvement in spiritual well‐being of both patients and their family caregivers was identified by Badger and colleagues (Badger et al. (2011) *p* < .01). Other studies identified significant improvement among survivors (Badger, Segrin, Pasvogel, et al. (2013) *p* < .01) or partners (Badger, Segrin, Hepworth, et al. (2013) *p* < .001). No significant change in spirituality was observed in three studies (Clark et al., 2013; Meyers et al., 2011; Mosher et al., 2018).

## 4 DISCUSSION

The catalyst for this review was the necessity to evaluate the characteristics and effectiveness of psychosocial interventions on QoL domains of adult people with cancer and their family caregivers particularly in developing countries. In our search, 12 psychosocial interventions were identified, but none had been conducted in developing countries despite the high burden of cancer care in those countries. Effectiveness of various psychosocial interventions on QoL was evaluated in RCT, two‐group and one‐group pre–post designs. As research in this area progresses more, rigorous designs such as RCT should be used to test refined and emerging interventions. Generally, the sample size of included studies was satisfactory, ranging from 80–968, with 3,390 patients/family caregivers overall. The psychosocial interventions were offered in a range of formats over a mean time of 8 weeks. Four of the interventions were nurse‐led, and nurses also participated in other interventions as part of a wider health team.

This review identified that nine studies used a theoretical framework which predominantly included the transactional stress‐coping model, CBT and interpersonal theory. The application of theory provides an understanding of the problem from a certain perspective, and informs the nature of the intervention and mechanisms underlying the anticipated improvement in outcomes (Rebok, 2013). A meta‐analysis by Prestwich et al. (2014) on effectiveness of health behaviour interventions found that interventions based on theory or theoretical constructs were more effective than those not using theory. Our review supports this with significant positive effects of theoretically based interventions on psychological, physical and social QoL outcomes over time. However, the impact of interventions on spiritual outcomes was often neglected or produced mixed results.

The application of interpersonal theory produced significant positive effects on all QoL domain outcomes over time. Interpersonal theory is highly relevant to interventions with adult patients/survivors of cancer and family caregivers given the focus on enhancing an individual's interactions with other people, especially significant others. The relevance of interventions based on interpersonal theory is relevant to spirituality, as such approaches can contribute to an improved sense of security and sense of self (Buechler, 2018). A previous study showed that interventions based on theories of interpersonal therapy for cancer patients and their family caregivers contributed to improvements in QoL (Badger, Segrin, Meek, Lopez, & Bonham, 2006). In addition, our findings support the conclusions of a review comparing interpersonal psychotherapy, supportive therapy and CBT by Evans (2009) which showed that interpersonal psychotherapy was most effective.

Although CBT is a beneficial therapy option for patients with various forms of cancer (Brothers, Yang, Strunk, & Andersen, 2011), the current review found either no significant improvement across QoL domains (Clark et al., 2013; Kayser et al., 2010) or a significant decline in psychological and social well‐being (Meyers et al., 2011). These results contradict those from a recent meta‐analysis which demonstrated the efficacy of CBT on QoL and psychological health of survivors/patients with breast cancer (Ye et al., 2018). Similarly, Solaimani Khashab, Ghamari Kivi, and Fathi (2017) reported a positive impact of group CBT on improving spiritual well‐being of bereaved persons.

In the current review, although three of the eight studies based on FOCUS, CBT and stress‐coping measured spiritual well‐being, none reported significant improvement. This could be because CBT was developed from empirical studies that did not consider faith as a variable (Carlson & Antonio, 2016). Similarly, it could be that CBT is individually focused and has less applicability than dyad‐based, interactional interventions for people with cancer and their family carers.

Regarding methods of delivery, the review found those interventions delivered by telephone had a positive effect compared with other methods of delivery. This result is comparable with that of Cox et al. (2017) who suggested that the impact of telephone interventions on outcomes was far greater than those using Internet delivery methods. In contrast, findings of our review differed from a recent systematic review and meta‐analysis of psychosocial interventions on the QoL of patients with colorectal cancer (Son et al., 2018), which highlighted that face‐to‐face intervention methods as compared with telephone‐based approaches had a significant effect on QoL. Given these various findings, further research on delivery of interventions is needed especially in developing countries where there may be limited access to telephone or Internet. It may be argued that face‐to‐face interventions appear to improve therapeutic relationships, thereby leading to an increase in patients'/caregivers' degree of adherence to treatment protocols and recommendations by healthcare providers (Bombard et al., 2018). In resource‐poor clinical environments, it may be prudent to use face‐to‐face methods as the key delivery component of psychosocial interventions for people with cancer and their family caregivers (Hurt, Walker, Campbell, & Egede, 2016).

The review found differences when reporting overall QoL compared with separate domains. Evaluating overall QoL without assessment of individual QoL domains could be misleading as cancer‐related distress occurs across all domains of QoL. For example, one study that reported a significant improvement on overall QoL reported no improvement in three of the four domains of patients/caregivers' QoL (Clark et al., 2013). A similar result may occur when studies are conducted in developing countries, whereby there may be little improvement in each domain due to poor medical resources and burden of care, but overall QoL may be significant. These conflicting results suggest the need to measure and report individual domains and overall QoL to ascertain the true effects of interventions.

The cultural context where QoL is measured is also a key issue. What is considered “a good life” varies between individuals and different societies and cultures. It may be misleading to take QoL concepts developed in one cultural context and apply them to other cultures or even in different ethnic communities (Zhang et al., 2016). Various communities/cultures may attribute different levels of importance to domains of QoL. For instance, spirituality is more profound in religious societies in sub‐Saharan Africa and developing countries in general. Although spirituality goes beyond religiosity (Arrey, Bilsen, Lacor, & Deschepper, 2016), it is the first stage towards spiritual development. In many African cultures, spirituality has an important role in coping, survival and maintaining overall well‐being following a cancer diagnosis (Arrey et al., 2016). It could be that spirituality optimizes functioning across the other QoL domains. The relationship between spirituality and psychosocial well‐being has been found to be essential to health (Laird, Krause, Funes, Lavretsky, & Lavretsky, 2019). However, our review found half the studies did not consider the spiritual domain of QoL and is similar to findings by other researchers (Hu et al., 2019). There needs to be a paradigm shift in theoretical approaches that would enable development of new psychosocial interventions to address spiritual well‐being alongside other QoL domains of people with cancer and their family caregivers.

### 4.1 Strengths and limitations

The strengths of this review include a rigorous literature search, use of a validated methodology and use of two independent reviewers during data evaluation, data extraction and synthesis. It is conceivable, however, that some articles may have been missed despite implementing a comprehensive and rigorous search strategy across key databases for published peer‐reviewed literature. Also, the large proportion of studies assessed as “weak” and narrative account of results limits the extent to which definitive statements or firm conclusions may be drawn from the review. Despite the scope of the review, all studies were conducted in developed countries. No study was published from developing countries highlighting the lack of cancer and QoL research (Ogunbiyi, Stefan, & Rebbeck, 2016). Most studies recruited participants from white ethnicities, ignoring the possibility that race, culture and beliefs may influence individuals' QoL. Therefore, the themes and conclusions are mainly representative of participants from those developed countries and may differ from those of the developing countries. Furthermore, many important studies that aimed to improve aspects relevant to QoL but did not measure this construct specifically were not included in the current systematic review. Finally, only half the included studies gave consideration to the spiritual well‐being of people with cancer and their family caregivers. This is an important gap in psychosocial intervention studies. It is possible that over time, more studies measuring spirituality will be available and results of future reviews may differ from this one.

## 5 CONCLUSION

This review identified the characteristics and effectiveness of psychosocial interventions on QoL of adult people with cancer and their family caregivers. Interventions were predominantly aimed at improving coping skills, communication and behaviour change to assist patients and their family to set short‐term goals, improve coping and reduce uncertainty. The highest benefit was gained from telephone interventions. The analysis of rigour and bias identified that most studies were characterized by weak methodology and quality. Therefore, we cannot draw firm conclusions about the effectiveness of psychosocial interventions on QoL domains among people with cancer and family caregivers. There is a need for rigorous research especially in developing countries. Findings from this review contribute to a deeper understanding of the psychosocial therapies and delivery modes of interventions. Future research should use well‐known outcome measures to maximize homogeneity and allow pooling of results.

## 6 RELEVANCE TO PRACTICE

This systematic review confirms evidence that psychosocial interventions offered to people with cancer and family caregivers can contribute to positive effects on important QoL outcomes. Although effects on QoL domains were mixed, the findings show number of potential implications for clinical practice, research and education. First, health workers need to be aware that people with cancer and family caregivers tend to respond to cancer and its treatment as a unit; hence, patient/family caregivers should be considered as a dyad when planning care protocols. The findings from this review and those of others (Aubin et al., 2017; Treanor. et al., 2019) suggest that adjustment to cancer is a family affair, not only because both patients and family carers have legitimate need for support, but also because role adjustment problems in the family will negatively affect the long‐term adjustment of the patient.

The review concluded that interventions based on interpersonal therapy were more effective than other therapies. It is important for healthcare professionals to promote a therapeutic relationship that encompasses caring and supportive behaviours towards patients and family caregivers. Clinician–patient/family caregiver relationships engender interactions characterized by effective communication and facilitate improved patient satisfaction, adherence to treatment, quality of life and decreased healthcare costs. Findings of the review may prompt greater awareness of nurses about the spiritual well‐being of cancer patients and their family caregivers. Promoting the integration of spiritual care in nursing care will require additional education and research to assist understanding, minimize confusion about the differences between spirituality and religion and develop effective nursing care practices. There is an urgent need for effective interventions to be replicated and tested in other locations, under different socio‐cultural conditions and according to different types and stage of cancer.

## CONFLICT OF INTEREST

The authors declare no conflict of interest.

## AUTHORS' CONTRIBUTIONS

IG, DC and EC: Study design and PRISMA guideline, and manuscript preparation; IG and EC: Database search, study selection, quality assessment and data extraction.

## ETHICAL APPROVAL

None.

## Supporting information

File S1
